# Automatic Apparent Nasal Index from Single Facial Photographs Using a Lightweight Deep Learning Pipeline: A Pilot Study

**DOI:** 10.3390/medicina61111922

**Published:** 2025-10-27

**Authors:** Babak Saravi, Lara Schorn, Julian Lommen, Max Wilkat, Andreas Vollmer, Hamza Eren Güzel, Michael Vollmer, Felix Schrader, Christoph K. Sproll, Norbert R. Kübler, Daman D. Singh

**Affiliations:** 1 Department of Oral, Maxillofacial and Facial Plastic Surgery, Medical Faculty and University Hospital Düsseldorf, Heinrich-Heine-University Düsseldorf, 40225 Düsseldorf, Germany; 2 Department of Oral and Maxillofacial Plastic Surgery, University Hospital of Würzburg, 97070 Würzburg, Germany; 3 Department of Radiology, University of Health Sciences, İzmir City Hospital, 35170 İzmir, Turkey; hamzaerenguzel@gmail.com; 4 Department of Oral and Maxillofacial Surgery, Tübingen University Hospital, Osianderstrasse 2-8, 72076 Tuebingen, Germany

**Keywords:** anthropometry, image processing, computer-assisted, deep learning, neural networks, computer, rhinoplasty, surgery, plastic, decision support systems, clinical, nose

## Abstract

*Background and Objectives*: Quantifying nasal proportions is central to facial plastic and reconstructive surgery, yet manual measurements are time-consuming and variable. We sought to develop a simple, reproducible deep learning pipeline that localizes the nose in a single frontal photograph and automatically computes the two-dimensional, photograph-derived apparent nasal index (aNI)—width/height × 100—enabling classification into five standard anthropometric categories. *Materials and Methods*: From CelebA we curated 29,998 high-quality near-frontal images (training 20,998; validation 5999; test 3001). Nose masks were manually annotated with the VGG Image Annotator and rasterized to binary masks. Ground-truth aNI was computed from the mask’s axis-aligned bounding box. A lightweight one-class YOLOv8n detector was trained to localize the nose; predicted aNI was computed from the detected bounding box. Performance was assessed on the held-out test set using detection coverage and mAP, agreement metrics between detector- and mask-based aNI (MAE, RMSE, R^2^; Bland–Altman), and five-class classification metrics (accuracy, macro-F1). *Results*: The detector returned at least one accepted nose box in 3000/3001 test images (99.97% coverage). Agreement with ground truth was strong: MAE 3.04 nasal index units (95% CI 2.95–3.14), RMSE 4.05, and R^2^ 0.819. Bland–Altman analysis showed a small negative bias (−0.40, 95% CI −0.54 to −0.26) with limits of agreement −8.30 to 7.50 (95% CIs −8.54 to −8.05 and 7.25 to 7.74). After excluding out-of-range cases (<40.0), five-class classification on n = 2976 images achieved macro-F1 0.705 (95% CI 0.608–0.772) and 80.7% accuracy; errors were predominantly adjacent-class swaps, consistent with the small aNI error. Additional analyses confirmed strong ordinal agreement (weighted κ = 0.71 linear, 0.78 quadratic; Spearman ρ = 0.76) and near-perfect adjacent-class accuracy (0.999); performance remained stable when thresholds were shifted ±2 NI units and across sex and age subgroups. *Conclusions:* A compact detector can deliver near-universal nose localization and accurate automatic estimation of the nasal index from a single photograph, enabling reliable five-class categorization without manual measurements. The approach is fast, reproducible, and promising as a calibrated decision-support adjunct for surgical planning, outcomes tracking, and large-scale morphometric research.

## 1. Introduction

Facial plastic and reconstructive surgery relies heavily on precise evaluation of nasal proportions for aesthetic planning and optimal outcomes [1]. The nose is the central and most prominent facial feature, and its dimensions (width, height, and angles) are key determinants of facial harmony [2]. Achieving a balanced profile in rhinoplasty or facial reconstruction requires careful assessment of nasal shape, as even subtle modifications can alter the overall facial aesthetics [3]. In the current clinical scenario, where demand for rhinoplasty is rising, a detailed and objective assessment of nasal dimensions has become a necessity for surgical planning [4,5].

One standard anthropometric index of nasal shape is the nasal index (NI), defined as the ratio of nasal width to nasal height (often multiplied by 100 in anthropological contexts) [6]. The NI provides a single number summarizing nose proportion, and it serves as the basis for classifying nose types into five morphological categories: hyperleptorrhine (very narrow nose, NI < 54.9), leptorrhine (narrow nose, NI 55.0–69.9), mesorrhine (medium, NI 70.0–84.9), platyrrhine (broad, NI 85.0–99.9), and hyperplatyrrhine (very broad, NI ≥ 100) [7]. Such classifications, originally developed in anthropometry, can be used by surgeons and anthropologists to describe nasal morphology in a quantitative way. However, obtaining these measurements in practice is often labor-intensive. Traditional anthropometry involves placing calipers on the patient or analyzing photographs manually, which is time-consuming and dependent on the examiner’s experience [8]. Soft tissue landmarks can be difficult to pinpoint accurately, and factors like tissue elasticity or patient movement introduce variability and subjectivity in measurements [8]. In short, the manual assessment of nasal index is prone to inconsistency, underscoring the need for an automated, objective approach.

Recent advances in computer vision and deep learning offer promising solutions for objective facial analysis. Research in facial anthropometry has increasingly leveraged deep learning models to detect landmarks and assess morphological parameters with high throughput and consistency [8]. Convolutional neural networks (CNNs) have been applied to tasks like facial feature localization and dimension measurement, enabling automated computations of indices and angles that were previously measured by hand [8]. For example, object detection models such as YOLO (You Only Look Once) have been used to localize facial structures as a first step in automatic craniofacial measurements [8,9]. These approaches can significantly reduce the time required for analysis and eliminate inter-observer variability, making them attractive for clinical use where reproducibility is critical. Nevertheless, to train such models for nasal index assessment, a sufficiently large and labeled dataset of nose shapes is required—something that has been lacking, since prior studies typically measured nasal indices on relatively small patient cohorts manually [6].

In this study, we address the above gap by leveraging an existing face dataset to create a novel pipeline for automatic nasal index classification. We utilize the CelebA dataset, a large-scale repository of high-resolution face images [10]. From these images, we compute the apparent Nasal Index (aNI) for each face—defined as the width-to-height ratio of the nose region as observed in the frontal 2D image. By applying the standard NI thresholds, we label each nose in the dataset with one of the five anthropometric categories (from hyperleptorrhine to hyperplatyrrhine). In essence, the dataset acts as a rich source of measurements, allowing us to assemble thousands of examples of each nasal category for training and evaluation of a classifier. Furthermore, we develop a fully automated pipeline that can estimate the nasal index from a raw image without requiring a pre-existing mask. To achieve this, we trained a lightweight one-class YOLOv8 model to detect the nose region in a frontal face image. YOLOv8, as an advanced real-time object detector, yields a bounding box around the nose, from which the width and height of the nose can be approximated. By integrating this detector with our aNI computation and classification scheme, we enable nasal index classification in a single step from an input face photograph. This means that even for new images (e.g., a patient’s preoperative photo) where no segmentation is available, the system can localize the nose, measure its apparent width and height, compute the aNI, and automatically determine the nose type category. The entire process—from nose detection to index calculation and category output—is automated and fast, requiring no manual intervention. Because our open-source model focuses on a single class (the nose), it remains lightweight and efficient, making it feasible for deployment in clinical software or mobile applications.

This automated pipeline has important implications for both clinical and research settings. In surgical planning, an objective tool for nasal index measurement can assist surgeons in quantifying a patient’s nasal proportions and tracking changes after reconstruction or rhinoplasty. It provides a consistent frame of reference (e.g., identifying if a nose falls within the “mesorrhine” normal range or towards the extremes), which can improve communication and planning of surgical goals. By removing subjective bias, the system may help standardize assessments and support decision-making with quantitative data. From a research perspective, the ability to classify nasal morphology at scale opens new avenues in morphometric analysis—for instance, analyzing correlations between nasal index and other craniofacial parameters or outcomes. Moreover, our use of a publicly available dataset and off-the-shelf deep learning architectures ensures that the method is reproducible and can be built upon by others. The pipeline could be integrated into educational tools for surgical training or into forensic analysis software wherever rapid nasal measurements are needed, all while maintaining consistency and accuracy. The aim of the study was to develop the end-to-end pipeline that can classify noses into standard anthropometric categories from a single image, thereby providing a simple and objective tool for nasal proportion assessment in clinical practice and morphometric analysis without manual measurements.

## 2. Materials and Methods

### 2.1. Data Source and Image Selection

No patient data were used; all images originate from publicly available online sources. We used face photographs from the CelebA [10] family of datasets and constructed a curated set of 29,998 high-quality, near-frontal images in which the external soft-tissue nose was clearly visible and not heavily occluded (e.g., by hands, microphones, or accessories). Images with marked yaw/pitch, severe blur, or substantial nasal occlusion were excluded during screening. Within the CelebA subset used for this study (n = 29,998), 57.7% of the images depicted female subjects (17,318/29,998) and 42.3% male subjects (12,680/29,998). Based on the Young attribute provided in the dataset, 77.9% (23,378/29,998) of the faces were labeled as young and 22.1% (6620/29,998) as not young, reflecting the known demographic skew of CelebA toward younger female individuals. The dataset does not include race/ethnicity labels and we did not infer them; thus, we cannot report race/ethnicity proportions. The curated images were randomly partitioned once with a fixed seed into training (20,998), validation (5999), and test (3001) sets in a 70/20/10 randomized ratio. All analyses were performed at the image level.

### 2.2. Nose Annotation and Mask Generation

Each image was annotated using the VGG Image Annotator (VIA) (VIA, University of Oxford, Oxford, UK) polygon tool to delineate the visible external nose. The annotation protocol defined the nose as the externally visible soft-tissue envelope including the alar wings and columella; polygons were drawn along the visible skin boundary in frontal view. VIA projects were exported and rasterized to binary nose masks (foreground = 1, background = 0) with the same spatial resolution as the corresponding image. Masks with zero foreground or obvious polygoning errors were discarded prior to split assignment to avoid leakage.

### 2.3. Ground-Truth Apparent Nasal Index and Categorical Labels

From each binary mask we derived an axis-aligned bounding box (x_min_, y_min_, x_max_, y_max_) by the extremal foreground pixel indices. We treat pixel coordinates as half-open intervals in the continuous image plane. Let

breadth_mask_ = (x_max_ + 1) − x_min_,

height_mask_ = (y_max_ + 1) − y_min_.

The apparent nasal index (aNI) was defined as the scale-invariant ratio

aNI_mask_ = 100 × breadth_mask_/height_mask_.

We assigned nasal categories using pre-specified thresholds applied identically throughout the study: hyperleptorrhine (40.0–54.9), leptorrhine (55.0–69.9), mesorrhine (70.0–84.9), platyrrhine (85.0–99.9), and hyperplatyrrhine (≥100.0). aNI reflects 2D appearance under the prevailing camera geometry and is not intended as a surrogate for true 3D anthropometry (e.g., it omits nasal depth/projection). We use ‘apparent’ throughout to avoid implying 3D anatomical measurement.

### 2.4. Nose Detector Model and Training

We trained a lightweight, one-class detector (YOLOv8-n) to localize the nose directly from RGB images so that nasal index can be inferred without a segmentation mask at inference. CelebA provides aligned face crops at 178 × 218 px, which we rescaled to 640 × 640 for YOLO input; all analyses were performed on the rescaled images. Training was performed for 50 epochs at an input size of 640 × 640, batch size 32, with early-stopping patience = 20 and a fixed random seed (12,345); determinism was enabled. Optimization followed the YOLO default configuration (stochastic gradient descent with initial learning rate 0.01, momentum 0.937, weight decay 5 × 10^−4^; warm-up for 3 epochs). Data augmentation used the built-in YOLO policy: horizontal flip probability 0.5, random scaling up to ±50%, translation up to 10%, HSV jitter (h = 0.015, s = 0.7, v = 0.4), and mosaic augmentation active except during the last 10 epochs. No rotation, shear, mixup, or cutmix were applied. The best checkpoint was selected by the validation objective provided by the framework. Training and inference were executed in Python 3.10 (Python Software Foundation, Wilmington, DE, USA) with PyTorch 2.5.1 (Meta Platforms, Inc., Menlo Park, CA, USA), CUDA 12.1 (NVIDIA Corporation, Santa Clara, CA, USA) and Ultralytics YOLO v8.3.179 (Ultralytics Ltd., London, UK) on a CUDA-enabled workstation running Windows 10 (Microsoft Corporation, Redmond, WA, USA).

### 2.5. Inference Pipeline and Predicted Nasal Index

For each test image, the detector produced zero or more nose candidates after non-maximum suppression (IoU threshold 0.7). We selected the highest-confidence detection (confidence threshold 0.1) and converted normalized box coordinates back to pixel space. Denoting the predicted pixel-space box by (x_1_, y_1_, x_2_, y_2_), the predicted apparent nasal index was

aNI_det_ = 100 × (x_2_ − x_1_)/(y_2_ − y_1_)

Predicted nasal categories were then obtained by applying the same thresholds used for ground truth.

### 2.6. Evaluation Metrics and Analysis

Detector performance was quantified on both validation and test sets using precision, recall, mAP@0.5, and mAP@0.5:0.95 (area under the precision–recall curve across IoU thresholds from 0.50 to 0.95 in 0.05 increments) computed by the YOLO evaluator with the aforementioned non-maximum suppression and confidence settings. We additionally reported detection coverage on the test set, defined as the proportion of images with at least one accepted nose detection (confidence ≥ 0.1).

Agreement between predicted and ground-truth nasal indices on the test set was assessed by the mean absolute error (MAE) in NI units, the root mean squared error (RMSE), and the coefficient of determination (R^2^) computed as the squared Pearson correlation between aNIdet and aNImask. Images without an accepted detection were excluded from aNI error calculations but are inherently reflected in the detector metrics and coverage. We estimated 95% CIs by nonparametric bootstrap with 2000 image-level resamples (seed = 12,345) for MAE and macro-F1; accuracy 95% CI used the Wilson method. Bland–Altman limits and CIs followed the standard normal-theory approach. LOESS curves used a span of 0.6 (statsmodels), shaded with bootstrap bands.

For categorical performance we compared mask-derived classes against detector-derived classes on the test set using overall accuracy, macro-averaged F1-score (unweighted mean of class-wise F1 across the five defined categories), and the confusion matrix. Consistent with the labeling policy, images with out-of-range nasal index (aNI < 40.0 in either reference, mask-derived or detector-derived) were excluded from the five-class metrics but their counts were reported. All statistics were computed once on the held-out test set with the fixed split; no cross-validation or model ensembling was performed. To quantify ordinal consistency, we computed weighted Cohen’s κ (linear and quadratic) and Spearman’s ρ between mask- and detector-derived classes. To summarize near-misses, we report adjacent-class (within-one-bin) accuracy. Robustness to threshold choice was assessed by shifting all anthropometric cut-points ±2 NI units and recomputing 5-class metrics. Attribute-level consistency was explored by repeating analyses after merging with CelebA attributes (Male, Young, coded 1/−1). Images with aNI < 40.0 in either axis remained excluded from categorical metrics, consistent with our main protocol. On the test set, non-accepted images (no detection above the confidence threshold) were 1/3001 (0.03%), corresponding to 99.97% detection coverage; additionally, 24/3000 (0.8%) of accepted detections were out-of-range (aNI < 40.0) and therefore excluded from five-class metrics.

## 3. Results

### 3.1. Nose Detection Performance

**Figure 1:**
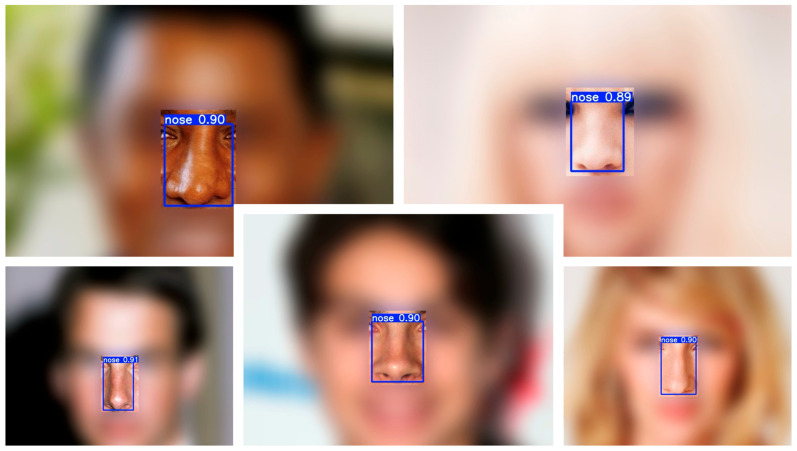
Example nose detections on CelebA test images. Representative cases with a single predicted nose bounding box (blue) and confidence score (white). The detector localizes the nose consistently across a range of nasal shapes and photographic conditions.

**Figure 2:**
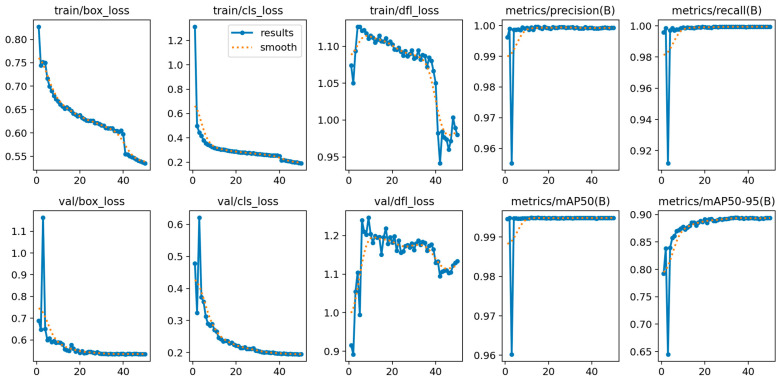
Training/validation dynamics of the one-class nose detector. Epoch-wise losses (box, classification, distribution—focal) and validation metrics (precision, recall, mAP@0.5, mAP@0.5:0.95). Curves demonstrate rapid convergence and a stable plateau by ~epoch 30.

Qualitative examples across a variety of facial appearances illustrate robust localization (Figure 1). A lightweight one-class detector (YOLOv8-n) localized the nose with near-perfect precision and recall on the validation set (precision ≈ 0.999, recall ≈ 0.999), achieving mAP@0.5 = 0.995 and mAP@0.5:0.95 = 0.894 at convergence. Training and validation curves showed rapid stabilization by ~epoch 30 and a flat plateau thereafter (Figure 2), indicating that the model had reached its performance ceiling without signs of overfitting. On the held-out test set, the detector achieved a precision of 0.999, recall of 0.999, mAP@0.5 of 0.995, and mAP@0.5:0.95 of 0.890, confirming near-perfect nose localization performance.

### 3.2. Agreement Between Detector-Based and Mask-Based Apparent Nasal Index

Among test images with an accepted detection (n = 3000), the detector-derived apparent nasal index (aNI_det_) closely tracked the mask-derived reference (aNI_mask_). The mean absolute error (MAE) was 3.04 NI units (95% CI 2.95–3.14), the RMSE was 4.05, and the coefficient of determination was R^2^ = 0.819. Because each category spans 15 NI units, the absolute error corresponds to ≈20% of a band, consistent with the predominance of adjacent-class swaps in the categorical analysis. The statement that MAE ≈ 3 NI equals ~20% of a 15-unit category width is a relative scale interpretation and does not imply a systematic 20% misclassification rate. Consistent with this small numerical error, misclassifications predominantly occur near decision thresholds and manifest as adjacent-class swaps (see Section 3.3) and quantified by the adjacent-class accuracy ≈ 100% and ordinal κ in Table 1.

**Figure 3:**
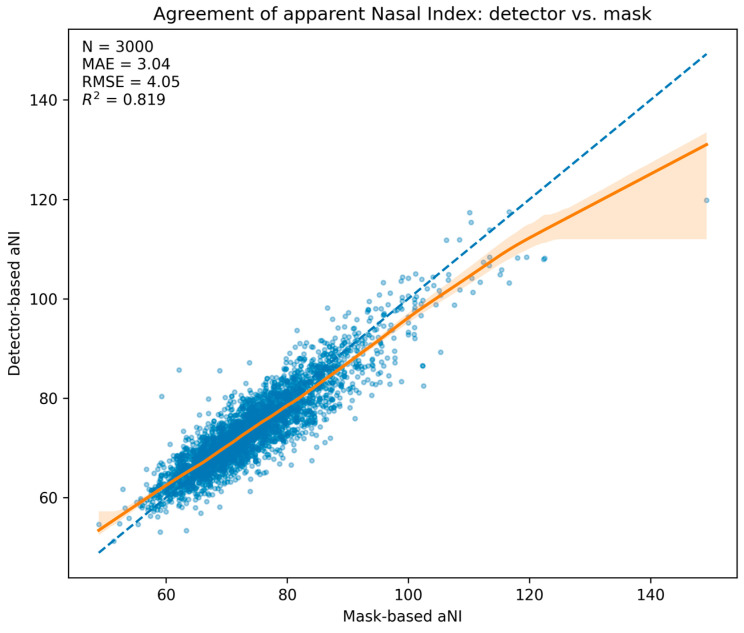
Agreement of apparent Nasal Index: detector vs. mask. Scatter of aNIdet versus aNImask for n = 3000 test images with LOESS trend (solid) and 95% bootstrap band (shaded); dashed line is identity. Embedded summary: MAE = 3.04, RMSE = 4.05, R2 = 0.819.

**Figure 4:**
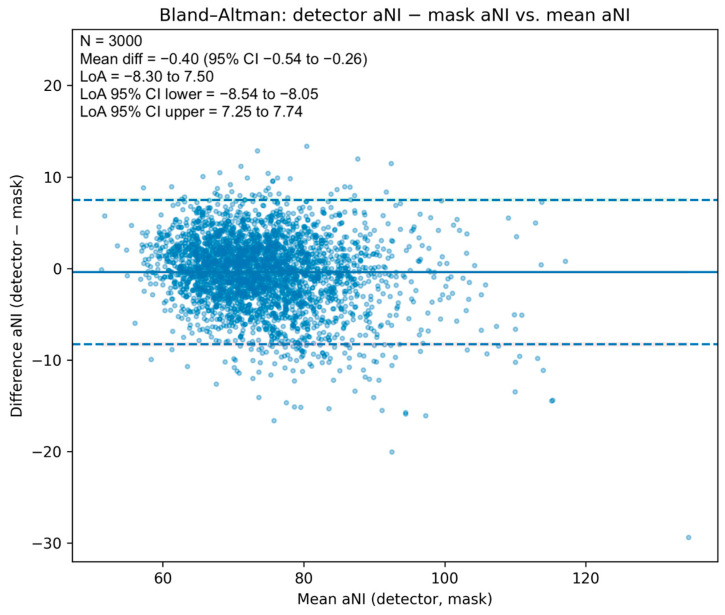
Bland–Altman plot for apparent Nasal Index. Differences (aNIdet − aNImask) vs. means, with mean bias (solid), limits of agreement (dashed), and 95% CIs for the mean and limits (shaded). Bias −0.40 NI units (95% CI −0.54 to −0.26); LoA −8.30 to 7.50.

The scatter with LOESS trend and 95% bootstrap band (Figure 3) shows close alignment with the identity line across the central range, with mild underestimation in the upper tail. A Bland–Altman analysis (Figure 4) yielded a mean difference of −0.40 NI units (95% CI −0.54 to −0.26), indicating a small negative bias of the detector relative to the mask reference, and limits of agreement of −8.30 to 7.50 NI units (95% CIs for the limits −8.54 to −8.05 and 7.25 to 7.74, respectively). Dispersion was approximately homoscedastic across most of the measurement range.

### 3.3. Five-Class Nasal Category Performance

**Figure 5:**
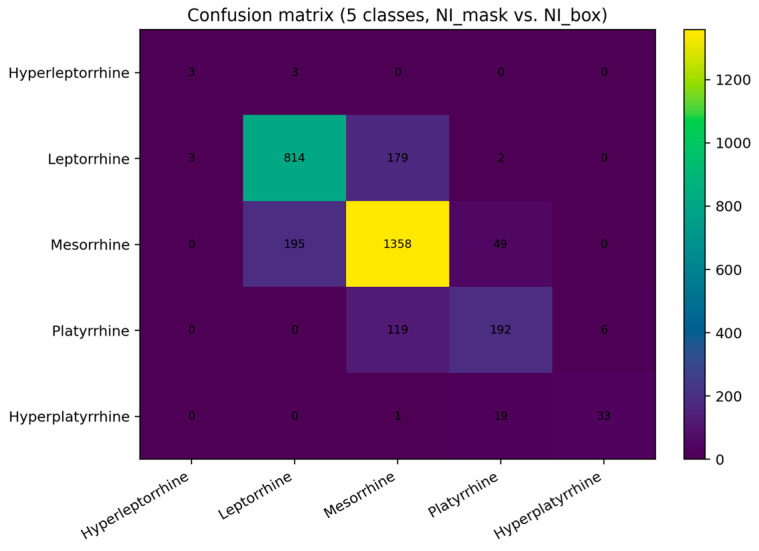
Confusion matrix for five nasal categories (test set). Rows: mask-derived classes; columns: detector-derived classes. Cell colors indicate count frequency, with higher values shown in yellow. The diagonal sums to 2400/2976 (80.7% accuracy). Off-diagonal counts are dominated by adjacent-class swaps (e.g., mesorrhine<->leptorrhine), consistent with borderline cases rather than gross errors.

We mapped both indices to five prespecified anthropometric classes using identical thresholds. Of the 3000 detected test images, 24 (0.8%) involved an out-of-range index (<40.0) on either axis and were excluded from five-class analysis, leaving n = 2976 comparisons. The detector reached a macro-averaged F1-score of 0.705 (95% CI 0.608–0.772) with an overall accuracy of 80.7% (2400/2976). Per-class recalls reflected both class prevalence and proximity to thresholds: leptorrhine 0.816 (814/998), mesorrhine 0.847 (1358/1602), platyrrhine 0.606 (192/317), hyperplatyrrhine 0.623 (33/53), and hyperleptorrhine 0.500 (3/6). The confusion matrix (Figure 5) shows that errors concentrate between adjacent categories—notably mesorrhine<->leptorrhine and platyrrhine<->mesorrhine—consistent with the small numerical differences around decision boundaries implied by the MAE.

**Figure 6:**
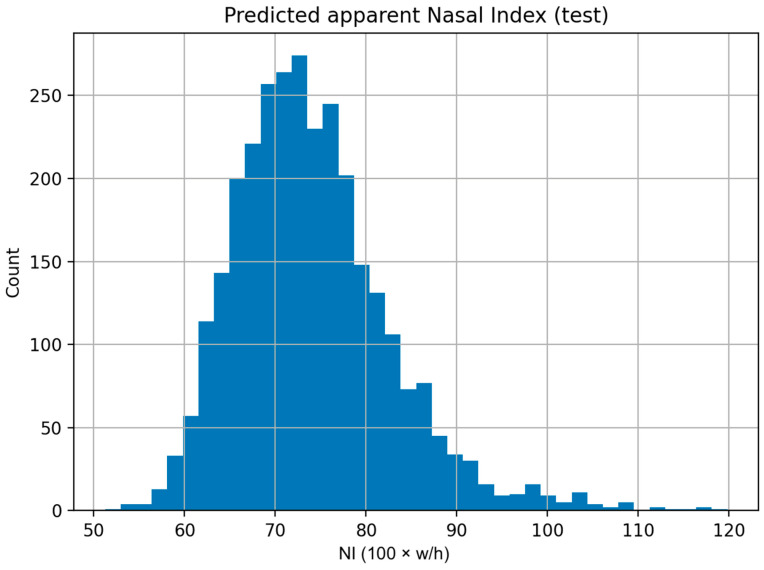
Distribution of detector-based apparent nasal index. Histogram of aNIdet = 100 × (box width/box height) for the 3000 detected test images. The unimodal shape with a modest right tail aligns with the mask-based reference distribution.

The distribution of detector-based aNI on the test set was unimodal with a mild right tail (Figure 6), mirroring the reference distribution and supporting stable behavior of the measurement across the typical range of nasal proportions.

### 3.4. Additional Agreement and Robustness Analyses

Weighted κ was 0.712 (linear) and 0.784 (quadratic), and Spearman’s ρ on ordinal class codes 0.763, confirming strong rank agreement. Adjacent-class accuracy was 0.999, indicating that nearly all misclassifications were borderline swaps between neighboring categories. Mean absolute error of aNI remained nearly the same with 3.06 units. Shifting all category thresholds ±2 NI units changed accuracy only marginally (−2 NI: 79.4%; +2 NI: 80.3%) and kept κ within 0.69–0.78, demonstrating robustness to cut-point selection. Stratified analyses showed similar performance across subgroups from CelebA attributes (Female 80.6%, Male 80.0%; Young 80.1%, Not Young 81.2%), with κ ≈ 0.71–0.81 and within-one-bin accuracy ≈ 1.0, supporting stability across sex and age.

## 4. Discussion

This study demonstrates that a lightweight deep learning pipeline can accurately and reliably estimate the nasal index (NI) from a single frontal photograph, achieving performance on par with manual measurements. The one-class YOLOv8n detector localized noses in virtually all test images (99.97% detection coverage), establishing a robust foundation for measurement. Predicted apparent NI values showed strong agreement with ground truth mask-derived NI, with a mean error of only ~3 NI units (MAE = 3.04, RMSE = 4.05) and high correlation (R^2^ = 0.819). In practical terms, this error is small—roughly 20% of one anthropometric category width—indicating that the model’s estimate typically falls within the correct nasal type or at most an adjacent category. Consistent with this, the five-class classification achieved an overall accuracy of 80.7% and a macro-average F1-score of 0.705. Misclassifications were largely limited to adjacent categories (e.g., mesorrhine vs. leptorrhine) rather than large errors. These results validate the efficacy of our automated pipeline: it not only detects the nose reliably but also provides an NI measurement with precision approaching that of manual methods. Agreement and robustness analyses further support the reliability of the approach. Ordinal metrics (κ ≈ 0.7–0.8, ρ ≈ 0.76) show that the detector preserves the rank order of nasal categories, and the almost-perfect adjacent-class accuracy (0.999) confirms that remaining errors are small borderline swaps rather than gross misclassifications. Stability of performance under ±2 NI threshold shifts and across sex and age subgroups indicates robust generalization within our curated near-frontal dataset.

Our findings are in line with, and extend, prior studies leveraging deep learning for facial anthropometry. Minh Trieu et al. (2023), for example, applied a convolutional neural network to measure multiple facial landmarks and distances on 2D images, reporting sub-millimeter accuracy in linear measures (mean error ~0.5 mm) [8]. Their success in automated photogrammetry underscores the feasibility of replacing tedious manual measurements with CNN-based algorithms, especially when multiple views or comprehensive landmark sets are used. Compared to their approach—which required three view angles and targeted numerous measurements—our method focuses on a single frontal view and a single composite metric (NI), yet still achieves high accuracy. This focus allows for a simpler, faster pipeline that could be more easily deployed in real-world settings. Similarly, Rao et al. (2019) explored machine learning for craniofacial measurements using a YOLO model to detect faces, followed by an active shape model to pinpoint facial landmarks [9]. While they attained 100% face detection and could identify many landmarks, their reported landmark errors ranged up to 4–6 mm [9], reflecting the challenges of limited training data (only 22 faces) and the two-step approach. In contrast, our end-to-end model was trained on nearly 30,000 faces, harnessing a large publicly available dataset to improve generalization. The resulting accuracy—with NI errors on the order of a few units (equivalent to only a few millimeters of linear deviation given typical adult nose dimensions)—represents a notable advancement in automatic nasal measurement. It addresses the gap highlighted by earlier works and reviews that lamented the labor-intensiveness and inconsistency of manual anthropometry. By achieving high throughput and consistency, our study confirms that modern object detectors like YOLO can overcome prior limitations when sufficient data and annotation are available. Precise anatomical landmarking remains the gold standard for surgical planning. Recent cadaveric work provides rigorous quantitative facial landmarks with clear clinical implications (e.g., measuring nasal and orbital indices on cadavers to guide surgeons [11]). Our aNI pipeline complements such efforts by offering instant, scalable 2D screening that can be calibrated to match caliper/3D or landmark-based measurements and extended toward derived indices like the facial index (FI) and orbital index (OI) in a modular fashion. However, our target clinical use is to support documentation, pre/post-operative tracking, and shared decision-making, not autonomous surgical decisions. Our approach complements recent rhinoplasty evaluation frameworks using CNNs by focusing on a rapid, single-view index that can be calibrated and tracked longitudinally [12]. It also aligns with evidence that web-based facial analysis tools can achieve acceptable agreement for rhinoplasty assessment when used as standardized adjuncts rather than definitive measurements [13].

From a clinical perspective, the implications of this automated NI estimator are encouraging. In rhinoplasty and reconstructive surgery, objective tools for analyzing nasal proportions can greatly aid preoperative planning and postoperative evaluation [12]. Surgeons currently often rely on calipers or visual assessment to judge nasal width and height, which is time-consuming and subject to inter-observer variability. Our system can instantly provide standardized measurements: for example, determining that a patient’s nose falls into the “mesorrhine” (average) category versus a “platyrrhine” (broad) category offers a concrete reference point for surgical goals. Such quantification can improve surgeon–patient communication; surgeons can explain planned changes in terms of NI units or category shifts, making abstract aesthetic concepts more tangible. It also enables tracking of surgical outcomes: an objective NI before and after rhinoplasty allows assessment of how much a nasal width or height was altered in relation to the face. Furthermore, the consistency of an algorithmic measurement (free from human bias or fatigue) may help standardize evaluations across clinics and practitioners. This is particularly useful in high-volume centers or in training settings, where having a “second pair of eyes” in the form of an AI tool can ensure no detail is overlooked. While the nasal index is a relatively simple descriptor, it encapsulates a key aspect of facial harmony; thus, its routine use via an automated pipeline could refine the precision of facial symmetry evaluations and aesthetic analyses in practice. For instance, if integrated into facial analysis software, our detector could be combined with other measurements (e.g., facial width or inter-pupillary distance) to yield a more complete picture of facial proportions and symmetry for each patient. For clinical fidelity, we envisage a two-stage external benchmark and calibration procedure. First, nasal width and height would be measured with calipers or 3D scans on a prospective convenience cohort (e.g., 30–50 clinic participants) to quantify the agreement between aNI (apparent nasal index) and the physical NI, and to learn a simple calibration mapping (for example, a linear or isotonic regression from aNI to true physical NI). Second, we would evaluate sex-stratified aNI threshold values and document any bias or variance across sex and age groups. This plan would effectively calibrate aNI as a reliable point-of-care surrogate for tracking pre- and post-operative nasal proportions against established normative curves, providing a path to integrate aNI measurements into individualized surgical decision support.

The reproducibility and scalability of our approach are also noteworthy. We built the model using an open dataset (CelebA) and standard deep learning frameworks, which means others can readily reproduce or adapt our pipeline. By releasing our code and relying on an off-the-shelf architecture, we align with open-science principles that facilitate external validation. This is in contrast to some prior works that used proprietary images or hardware, hindering replication. A practical advantage of our model is its efficiency: YOLOv8n is a lightweight network, enabling real-time inference on common hardware. This opens the door to deployment in various settings—potentially as a mobile app or a plug—in to electronic medical record systems—without the need for specialized equipment. The use of apparent NI derived from 2D photos also means data collection is simple: any standard frontal facial photograph (even taken by a smartphone) could be analyzed, lowering the barrier to large-scale studies. In research, this capability allows scholars to process thousands of images for morphometric analysis quickly, something not feasible with manual methods. For example, epidemiological studies could examine correlations between nasal index and demographic or clinical variables across large populations (while our study deliberately avoids any ethnic labels or biases, such analyses could be done in a controlled, ethical manner) [14]. Furthermore, our pipeline could serve as an educational tool: students and surgical trainees might use it to objectively compare their manual measurements with the AI’s output, honing their skills in identifying facial landmarks. In forensic science and anthropology, where rapid characterization of facial features can aid in identifying individuals or assessing remains, an automated NI classification could be one component of a toolkit for profiling facial morphology. The consistent and fast output of our method is advantageous in any scenario requiring high-throughput, repeatable facial measurements. Finally, functional restoration is a central objective in nasal surgery alongside aesthetics [15,16]. aNI quantifies a 2D proportion (width/height) and does not capture airway patency or nasal resistance. In prospective deployment, we plan to report aNI in parallel with functional endpoints (e.g., objective airflow tests or validated patient-reported symptoms) so that aesthetic proportion and function can be evaluated together during planning and follow-up. This combined reporting would clarify the role of aNI as a complementary morphological marker rather than a surrogate for nasal function.

Despite these promising results, several limitations must be acknowledged. First, our system operates on 2D images and calculates an apparent nasal index rather than a true 3D anatomical measure. Photographic perspective and head pose can influence the observed width-to-height ratio of the nose [17]. Because aNI is 2D and box-based, it is susceptible to modest bias from pose, perspective, and lens distortion. Our curated near-frontal dataset reduces but does not eliminate these effects. We therefore present aNI as a fast, standardized index for documentation and research at scale, with planned clinical calibration to physical or 3D measures before treatment-critical decisions. We mitigated extreme cases by curating the dataset (excluding images with significant yaw or pitch), but minor pose variations still occur and could introduce error. The absence of an explicit pose correction step means the model assumes near-frontal images; in practice, a patient’s photo with even moderate rotation might yield a slightly erroneous NI. Future work should incorporate pose normalization or multi-angle imaging to capture the nose’s dimensions more authentically [18]. Relatedly, because we use 2D pixel measurements, our NI does not account for nasal depth or projection—characteristics that a plastic surgeon might consider (e.g., a high nasal bridge or a bulbous tip might not affect the 2D NI but are clinically relevant) [13]. Utilizing stereo imaging or inferring 3D shape from the frontal view (via morphable models or deep depth estimation) could enhance the pipeline to measure true anatomical nasal indices and other shape parameters. A second limitation is that our categorical classification approach did not use any per-class weighting or specialized handling of class imbalance. The five NI categories were taken as is, and because extreme categories (hyperleptorrhine, hyperplatyrrhine) are relatively rare in our data, the model had few examples of these during training. This likely contributed to the lower recall for those classes (e.g., only 50% for hyperleptorrhine in our test set). In future iterations, we could address this by augmenting underrepresented categories or employing a hybrid strategy (for instance, a regression network fine-tuned with a classification head that is balanced). It may also be beneficial to treat NI prediction as a regression task with a subsequent flexible thresholding, or even as a direct ordinal classification to penalize large misorders more than near-misses. Third, while using CelebA gave us ample data, it is an in-the-wild dataset of celebrity images that may not perfectly represent clinical photo conditions. Factors like consistent camera distance, lighting, and patient demographics in a clinical setting could differ [19]. We did not explicitly test the pipeline on a set of actual patient photographs; thus, real-world validation is warranted. Initial use in a controlled clinical environment (with standard frontal face photos taken pre- and post-operatively) would help ensure the tool’s measurements align with those obtained by surgeons in practice. Additionally, our ground truth for validation was derived from manual mask annotations on the same 2D images (apparent measurements), rather than physical anthropometric measurements. While this internal reference is appropriate for method development, an ideal validation would compare our automated NI to caliper-based NI or 3D scan measurements on a set of subjects [20]. Such a comparison would quantify how much “apparent NI” deviates from “actual NI” and confirm the clinical meaningfulness of our metric. Lastly, our focus was intentionally narrow—on the nasal index alone. This provides a clear proof-of-concept, but noses are complex 3D structures, and patients and surgeons are often concerned with other features (bridge shape, tip projection, symmetry of nostrils, etc.). Our pipeline could be expanded to detect additional nasal landmarks (e.g., alar base points, nasion, subnasale) to compute other standard measurements and even detect asymmetry. We see this single-index model as a stepping stone toward a more comprehensive automated facial anthropometry system.

In summary, these limitations suggest several avenues for future work. Incorporating pose-invariant techniques or multi-view imagery would allow more accurate and robust application of the pipeline in non-ideal conditions. Integrating our detector with 3D reconstruction methods could transform the current 2D index into a true spatial measurement, bridging the gap between photographic analysis and physical anthropometry. Improving the classification balance (through advanced training strategies or additional data) will likely enhance performance on rare nasal types, which is important if the tool is to be universally applicable. Moreover, a prospective clinical study deploying this tool in practice—for example, in a rhinoplasty clinic—could provide valuable feedback on usability and accuracy, as well as highlight any unforeseen issues (such as consistency of results across different ethnic groups or imaging devices). We anticipate that collaboration with clinicians in testing the pipeline on patient photos will be a crucial step before full adoption. We do not infer patient ethnicity from images, nor do we endorse any physiognomic interpretation of facial features. It is recognized that face-image datasets can embed sampling biases; therefore, we include (i) stratified error reporting based on available attributes that serve as proxies for image quality or demographics (for example, evaluating performance on subsets with blurriness or occlusions), and (ii) a plan to audit errors across key subgroups (sex, age, imaging device) during clinical validation. Importantly, the tool is intended as a decision-support aid rather than a standalone diagnostic. Any clinical deployment will require human oversight, calibration against physical or 3D reference measurements, and explicit disclaimers about the tool’s appropriate use and limitations. We avoid normative claims about “ideal” facial features and commit to continuous fairness audits as the system is tested in diverse populations. The pipeline’s design is modular. For instance, one could replace the current single-class nose detector with a lightweight multi-landmark detection head (identifying key points such as the alar base, subnasale, nasion, etc.), enabling direct computation of additional anthropometric indices like FI and OI. Standard face-alignment techniques could normalize pose for these measurements. This extension would transform the present aNI-only proof of concept into a more comprehensive craniofacial analysis suite, capable of providing a range of facial indices (e.g., facial index, orbital index) and landmark-based measurements. Such a system would be well-suited for richer pre- and post-operative assessments, further supporting surgical planning and outcome tracking in a modular, extensible fashion.

## 5. Conclusions

We have developed an objective, automated method for nasal index estimation and classification using deep learning. Our one-step pipeline—enabled by a YOLOv8n nose detector—achieves high accuracy in measuring the apparent nasal index from frontal photos and reliably categorizes nose types into standard anthropometric classes without any manual intervention. This approach addresses the longstanding need for quick and reproducible nasal measurements in clinical and research settings. By leveraging a large public dataset and open-source tools, we ensured the solution is reproducible and adaptable. The results indicate that such an AI-driven tool can match human-level assessments of nasal proportions, offering consistent measurements that can enhance surgical planning, outcomes tracking, and large-scale morphometric studies. In conclusion, our study highlights a positive step toward integrating AI for facial anthropometry—providing surgeons and researchers with a practical, accurate instrument for nasal analysis. With further refinement and validation, this pipeline can be expanded and integrated into clinical workflows and educational platforms, ultimately contributing to improved patient care and a deeper understanding of facial morphology. The outlook is optimistic that automated facial measurement will become a standard adjunct in both clinical practice and research, combining efficiency with objectivity to benefit practitioners and patients alike.

## Figures and Tables

**Table 1:** Agreement, robustness, and stratified performance analyses on the held-out test set. Five-class metrics exclude images with apparent nasal index (aNI) < 40.0 on either axis, per protocol; counts shown as n (valid for 5-class).

Analysis Subset	n (Valid for 5-Class)	Accuracy [%]	Macro F1	Adjacent-Class Accuracy (±1 bin) [%]	Weighted κ (Linear/Quadratic)	Spearman ρ (Class Codes)	MAE (aNI Units)
Baseline (thresholds as defined)	2976	80.7	0.72	99.9	0.71/0.78	0.76	3.04
Thresholds − 2 NI units	2976	79.4	0.70	99.8	0.69/0.77	0.75	3.06
Thresholds + 2 NI units	2976	80.3	0.71	99.9	0.71/0.78	0.76	3.06
Sex stratification							
Female	1747	80.6	0.66	99.9	0.72/0.79	0.76	3.04
Male	1254	80.0	0.80	99.9	0.71/0.78	0.76	3.09
Age stratification							
Young	2361	80.1	0.72	99.9	0.71/0.78	0.76	3.05
Not Young	639	81.2	0.62	100.0	0.74/0.81	0.77	3.09

Abbreviations: aNI—apparent Nasal Index; κ—Cohen’s kappa; MAE—mean absolute error. Notes: (a) Adjacent-class accuracy (±1 bin) counts predictions within one neighboring anthropometric bin of the ground truth as correct. Because each category spans ≈ 15 NI units and the mean absolute error is ≈ 3 NI, most residual errors fall into an adjacent bin by design. (b) CelebA attributes “Young” and “Male” are binary dataset-level annotations (1/−1) used here as simple proxies for age and sex; they are not clinical labels. (c) Total detected test images = 3000; 24 images (0.8%) had aNI < 40.0 on either axis and were excluded from the 5-class metrics (valid n = 2976). (d) Per-class recall for rare extremes remains lower (see Section 3.3).

## Data Availability

We used an online dataset (CelebA) which is available for non-commercial research purposes from the Multimedia Laboratory of the Chinese University of Hong Kong, China. These imaging data are available from https://mmlab.ie.cuhk.edu.hk/projects/CelebA.html (accessed on 25 August 2025). The Python code and algorithm structures are available from: https://github.com/Freiburg-AI-Research (accessed on 25 August 2025).
